# Hybridization of Russian Sturgeon (*Acipenser gueldenstaedtii*, Brandt and Ratzeberg, 1833) and American Paddlefish (*Polyodon spathula*, Walbaum 1792) and Evaluation of Their Progeny

**DOI:** 10.3390/genes11070753

**Published:** 2020-07-06

**Authors:** Jenő Káldy, Attila Mozsár, Gyöngyvér Fazekas, Móni Farkas, Dorottya Lilla Fazekas, Georgina Lea Fazekas, Katalin Goda, Zsuzsanna Gyöngy, Balázs Kovács, Kenneth Semmens, Miklós Bercsényi, Mariann Molnár, Eszter Patakiné Várkonyi

**Affiliations:** 1 National Agricultural Research and Innovation Centre, Research Institute for Fisheries and Aquaculture, H-5540 Szarvas, Hungary; mozsar.attila@haki.naik.hu (A.M.); fazekas.gyongyver@haki.naik.hu (G.F.); farkas.moni@haki.naik.hu (M.F.); fazekas.dorottya.lilla@haki.naik.hu (D.L.F.); fazekas.georgina@haki.naik.hu (G.L.F.); 2 Festetics György Doctoral School, Georgikon Faculty, University of Pannonia, H-8360 Keszthely, Hungary; miklosbercsenyi77@gmail.com; 3 Juhász-Nagy Pál Doctoral School of Biology and Environmental Sciences, University of Debrecen, H-4031 Debrecen, Hungary; 4 Animal Husbandry Science Doctoral School, Szent István University, H-2100 Gödöllő, Hungary; molnar.mariann@hagk.hu; 5 Department of Biophysics and Cell Biology, Faculty of Medicine, University of Debrecen, H-4032 Debrecen, Hungary; goda@med.unideb.hu (K.G.); gyongy.zsuzsanna@med.unideb.hu (Z.G.); 6 Doctoral School of Molecular Cell and Immune Biology, University of Debrecen, H-4032 Debrecen, Hungary; 7 Department of Aquaculture, Institute for Conservation of Natural Resources, Faculty of Agricultural and Environmental Sciences, Szent Istvan University, H-2100 Gödöllő, Hungary; Kovacs.Balazs@mkk.szie.hu; 8 School of Aquaculture and Aquatic Sciences, Kentucky State University, Frankfort, KY 40601, USA; ken.semmens@kysu.edu; 9 Department of Animal Science, Georgikon Faculty, University of Pannonia, H-8360 Keszthely, Hungary; 10 National Centre for Biodiversity and Gene Conservation, Institute for Farm Animal Gene Conservation, H-2100 Gödöllő, Hungary; varkonyi.eszter@hagk.hu

**Keywords:** ploidy level, morphology, chromosome number, paternal haploid cell lineage, sturgeon

## Abstract

Two species from the families Acipenseridae and Polyodontidae, Russian sturgeon (*Acipenser gueldenstaedtii*, Brandt and Ratzeberg, 1833; functional tetraploid) and American paddlefish (*Polyodon spathula*, Walbaum 1792, functional diploid) were hybridized. The hybridization was repeated using eggs from three sturgeon and sperm from four paddlefish individuals. Survival in all hybrid family groups ranged from 62% to 74% 30 days after hatching. This was the first successful hybridization between these two species and between members of the family Acipenseridae and Polyodontidae. Flow cytometry and chromosome analysis revealed two ploidy levels in hybrids. The chromosome numbers of the hybrids ranged between 156–184 and 300–310, in “functional” triploids and “functional” pentaploids, respectively. The hybrid origin and the ploidy levels were also confirmed by microsatellite analyses. In hybrids, the size and the number of dorsal and ventral scutes correlated with the ploidy levels as well as with the calculated ratio of the maternal and paternal chromosome sets. An extra haploid cell lineage was found in three hybrid individuals irrespective of the ploidy level, suggesting polyspermy. Although the growth performance showed high variance in hybrids (mean: 1.2 kg, SD: 0.55), many individuals reached a size of approximately 1 kg by the age of one year under intensive rearing conditions.

## 1. Introduction 

In some fish taxa, polyploidization is an ongoing process and can occur, even within a species (autopolyploidy; i.e., polyploidy derived from a single species), while in other taxa, e.g., Cyprinidae, it is rare [1]. Polyploidy is relatively frequent in interspecific hybrids of fishes (allopolyploidy; i.e., polyploidy arising from interspecific hybridization). Berrebi [2] and Birstein et al. [3] pointed out that polyploidy increased the success of interspecific hybridization in fishes. 

Sturgeons represent ancestral ray-finned fishes (besides bichirs and ropefishes) in the class Actinopterygii [4], originating during the Jurassic era 200 million years ago [5,6]. The order Acipenseriformes consists of 27 living species, many of which are listed in the Appendices to CITES (Convention on International Trade in Endangered Species of Wild Fauna and Flora; [7]). They diverged before the teleost specific genome duplication from the other ray-finned fishes [8]. The sturgeons split into three families, one extinct and two presently living—Acipenseridae and Polyodontidae [9]. In the family Polyodontidae, no further genome duplication has occurred, and they are considered “functional diploids” (2n) with around 120 chromosomes. There are only two species in the family: American paddlefish (*P. spathula*), Chinese paddlefish (*Psephurus gladius*). The extinction of the Chinese paddlefish has been recently reported [10]. Several polyploidization evens occurred later in Acipenseridae, which can contribute to the resilience and adaptability of species belonging to Acipenseridae. Due to these evolutionary processes, as a byproduct, hybridization can be successful between Acipenseridae, and even among taxa with different chromosome numbers. In Acipenseridae, at least one additional genome duplication happened during the evolution of the Atlantic clade of the family, and more during the shortnose sturgeon (*Acipenser brevirostrum*) and the pacific clade evolution. Most of the species in the Atlantic clade, including the Russian sturgeon (*A. gueldenstaedtii*), are considered “functional tetraploid“ (4n) with ≈250 chromosomes [11]. 

Although hybridization among acipenserid species is common, there are no reports of successful hybridization of acipenserids and polyodontids. Previous hybridization experiments on shovelnose sturgeon (*Scaphirhynchus platorynchus*) × American paddlefish [12,13] or American paddlefish × Amur sturgeon (*Acipenser schrenckii*) [14] have failed to result in viable offspring. Besides the large phylogenetic distance (i.e., they diverged 184.4 Mya [15]), representatives of Polyodontidae and Acipenseridae differ in their gross morphology (e.g., presence of scutes, the structure of mouth, rostrum, filter apparatus) as well as feeding behavior, preferred habitat, etc. [3,16]. This suggests an inability to hybridize. In hybrids of acipenserids, odd ploidy plasticity may occur, which is highly dependent on the ploidy level of the parental species. Various ploidy levels are caused either by polyspermy or partial or full duplication of the maternal genome and hybridization [7,17]. Mosaicism also may occur [18,19,20]. 

During an experiment to produce gynogenic Russian sturgeon progeny, a negative control was initiated using non-irradiated American paddlefish sperm and eggs from the Russian sturgeon. Unexpectedly, the control cross resulted in viable hybrids. This was the first report of viable hybrids between any acipenserid and *Polyodon* species. In this study, we described the morphology, ploidy level, and chromosome number, demonstrating the hybrid origin of the progeny. This study addressed whether the simultaneous genome duplication occurs and how it affects the ploidy level of hybrid progenies. Finally, it described the relationship between the external morphology and ploidy level in the hybrids of species with such great phylogenetic distance. 

## 2. Materials and Methods

### 2.1. Reproduction and Nursing

Breeders originated from stocks that have been raised for three generations or more in captivity at the live gene bank, Szarvas, Hungary. For gynogenesis, we used irradiated paddlefish sperm. As a control of the success of gynogenesis, we ran parallel fertilization using untreated American paddlefish sperm. For the controls, three females from Russian sturgeon (tag codes: 6F15, 1748, and AG03) and four males from American paddlefish (tag codes: 2E75, 596B, 551F and 6517) were crossed and produced five parent hybrid combinations (Table 1). Experiments were ethically approved and reviewed by the Institutional Animal Care and Use Committee of Research Institute of Aquaculture and Fisheries (permission number NAIK/4842-1/2019, license number BE/25/4302-3/2017).

The breeders were injected with LHRH (luteinizing hormone-releasing hormone) analog Des-Gly^10^ (D-Ala^6^) LHRH-ethylamide (40 µg/kg for females and 20 µg/kg males) to induce ovulation and spermiation. Milt was stripped 48 h post injection, while eggs were stripped 48–52 h post injection using the method of Podushka, cited by Štěch et al. [21]. Immediately before fertilization, milt was diluted with water at a ratio of 1:200, and 2 mL of this diluted solution was added to egg samples weighing 300 g, according to the common aquaculture practice [22]. In the case of gynogenic fertilization, the milt was irradiated by 1 kGy dose of ^60^Co γ ray. For retention of the second polar body, a 2 min 36 °C heat shock was applied 18 min post fertilization [12,23]. Fertilization rates at 48 h post fertilization and survival at hatching were determined by counting 3 × 100 eggs in each parent combination. Two thousand newly hatched fry from each of the treatment groups were stocked into 400 L volume tanks with trough-flow water for nursing. On the eighth day after hatching, fry received frozen brine shrimp nauplii, and mosquito larvae followed dry feed feeding from the twentieth day. When fish reached the size of 3 cm, they were transferred into larger tanks. When fingerlings reached 12–15 cm, individuals were labeled with passive induced transponder (PIT) tags (Loligo® Systems, Toldboden, Viborg, Danemark).

### 2.2. Microsatellite Marker Analysis

Fin clips were collected from the female Russian sturgeons (6F15; 174B; AG03) and male American paddlefish (596B; 6517; 2E75) parents and from 19 hybrid offspring. Genomic DNA was extracted using the standard protocol of the E.Z.N.A. Tissue Kit (Omega Bio-Tek, Doraville, CA, USA). Four pairs of microsatellite primers were used. Three microsatellite loci (Psp-28, Psp-29, Psp-32) were developed for American paddlefish [24], and the fourth locus (Spl_101) was developed for shovelnose sturgeon [25]. The universal fluorescent labeling method was used for capillary electrophoretic analysis of microsatellite alleles [26]. The forward primers were tailed by a 5′ 17 bp long sequence to provide an attachment site for fluorescent dye-labeled (fluorescent dyes: FAM, VIC, NED, PET) “universal” oligos. One tail-specific oligo was added to each reaction mixture. The reactions consisted of 100 ng genomic DNA, 10× Dream Taq PCR buffer (it contains 20 mM MgCl_2_), 0.4 µL (0.2 mM of each dNTP), 0.5 µL forward and reverse primer, (10 µM), 0.5 µL fluorescent dye-labeled tail specific oligo (5′dye-ATTACCGCGGCTGCTGG-3′) (10 µM), and 1.0 U DreamTaq DNA polymerase in a total volume of 20 µL. PCR conditions: Initial denaturing at 94 °C for 2 min; 35 cycles of 94 °C denaturing for 30 s, annealing temperature (Table 2) for 30 s, 72 °C extension for 1 min, and a final extension at 72 °C for 5 min. Amplified, labeled products were subjected to capillary electrophoresis on an ABI Prism 3130 Genetic Analyzer (Applied Biosystems, Waltham, MA, USA). GeneMapper 4.0 Software (Applied Biosystems, Waltham, MA, USA) was used to determine the fragment size of the detected alleles.

### 2.3. Flow Cytometry and DNA Content Analysis

The DNA content of red blood cells (RBCs) was measured by flow cytometry using propidium iodide (PI) staining. Blood cells were centrifuged at 500× *g* for 5 min and re-suspended in 500 µL PBS to set the cell concentration to 10^7^/mL. Then, by adding 4500 µL 70% ethanol (Scharlab Hungary, Budapest) to the samples, the cells were fixed and permeabilized for 30 min at room temperature [27]. Subsequently, the samples were washed twice in PBS (at 500× *g* for 5 min and re-suspended in 5000 µL PBS). At that point, the ethanol-fixed cells were stained with 40 µg/mL PI (Sigma-Aldrich) for 30 min. The samples were measured by using a Becton Dickinson FACS Array flow cytometer. A green 532 nm laser was used for the excitation of PI, and the fluorescence signal was detected at 585/42 nm. Cell debris was excluded from analysis, on the basis of forward scatter (FSC) and side scatter (SSC) signals. Flow cytometry data were analyzed using the Flowing Software (Flowing Software 2.5.1 (released 4.11.2013)), Cell Imaging Core, Turku Centre for Biotechnology (Turku, Finland).

Flow cytometry measurements were carried out on 98 hybrid fish, two Russian sturgeon (6F15, Ag03), and four male American paddlefish (2E75, 551F, 596B, 6517). Each measurement was repeated three times independently. The analysis was carried out in a blind manner except for the four male American paddlefish blood samples. Upon DNA content analysis, the mean PI fluorescence intensity of each progeny was normalized to the mean PI fluorescence intensity measured in the case of the three male American paddlefish and expressed in pg.

The genome size of each individual was calculated using a modified formula by Tiersch et al. [28]: DNA content (pg) = 3.9× (I/P), where I and P are the fluorescence intensities of hybrids and paternal individuals, respectively. The constant of 3.9 is the DNA content of paddlefish cells given in the picogram [28].

### 2.4. Chromosome Analysis

Chromosome analysis was carried out on tissue culture cells, modified after Tóth et al. [29], from eight ten-month-old hybrid individuals. The fibroblast culture was set up from the pectoral fin. The culture medium was changed twice a week. After 4–6 weeks, when the fibroblast monolayer was almost confluent in the flasks (Greiner 690175; 50 mL), 40 µL of KaryoMAX™ Colcemid™ Solution (Gibco 15212012) was added. Two hours later, cells were trypsinized, and after 7-min centrifugation (1500× *g*) were resuspended in 0.56% KCL as hypotonic treatment; this was followed by three changes of methanol/acetic acid (3:1) fixative. The cell suspension was spread on slides, dried at room temperature, and stained with 5% fresh Giemsa solution (in phosphate buffer pH 7.0) (Sigma 48900) for 7–8 min [30]. Five slides from each individual were prepared, and at least 30 metaphase spreads per individual were examined to determine the chromosome number. Chromosomes were classified using the nomenclature of Levan et al. [31].

### 2.5. Morphometry 

After clove oil narcotization, we measured 21 morphometric and four meristic characters (Table 3) on 218 American paddlefish × Russian sturgeon hybrids, 49 American paddlefish, and 50 Russian sturgeon individuals with a digital caliper. The morphometric characters were determined according to Holcik et al. [32], Keszka and Krzykawski [33], and Hoover et al. [34]. In addition, we measured the width of the first three dorsal scutes. The meristic characters covered the number of dorsal (sDL), lateral (sLL), ventral scutes (sVL) and the number of barbels. The age of the examined fish varied between 228 and 258 days. 

Morphometric data were standardized to eliminate the effect of body size, according to Elliott et al. [35]. Fishes were divided into five groups based on their origin and genome size: American paddlefish (PF), Russian sturgeon (RS), small genome size hybrids (SH), large genome size hybrids (LH), and hybrids without genome size data (NH). We ran a series of non-parametric univariate tests (Kruskal–Wallis) and pairwise comparisons (Mann–Whitney U test with Bonferroni correction) for each morphometric and meristic character to reveal differences between groups. Principal component analysis (PCA) was performed on the entire standardized dataset to evaluate the morphometric variation. Scute size was excluded from the multivariate test because it was absent in all paddlefishes and many hybrids. Statistical analyses were performed using PAST 1.95 [36]. 

## 3. Results

### 3.1. Fertilization, Survival, Growth of Hybrids

The fertilization rate varied from 86 to 93% and the hatching rate from 78 to 85% in the five parent combinations. Survival after hatching at 30 days ranged from 62 to 74% and at 180 days from 49 to 68%. Substantial differences in individual weights were observed within the groups. By the end of the first year, many hybrid individuals reached a bodyweight of over 1 kg (mean: 1.2 kg, SD: 0.55). 

### 3.2. Microsatellite Analysis

There was no common allele between the two species. The Psp-28, Psp-32, and Spl-101 were disomic, while the Psp-29 was tetrasomic on the American paddlefish males. In the case of American paddlefish, all of the detected alleles of the four loci were different from the Russian sturgeon: five at the 2E75 males, six at the 596B males, and seven at 6517 males. Four alleles were common in both American paddlefish males (Table 4).

The Psp-29 was disomic, while the Psp-28, Psp-32, and Spl-101 were tetrasomic on Russian sturgeon females. Two female genotypes did not differ from each other. Altogether twelve alleles were detected at the four loci, and none of them were present in American paddlefish. All the markers segregated in the offspring groups. All hybrid individuals inherited one paternal allele from the disomic (Psp-28, Psp-32, and Spl-101) and two alleles from the tetrasomic alleles loci. The SH individuals inherited one maternal allele from the Psp-28 or Psp-29 and two alleles from the tetrasomic Psp-32 and Spl-101 loci, while the LH hybrids inherited two, three, or four alleles in the case of tetrasomic markers, but at least one of the two maternal tetrasomic loci revealed the presence of two maternal genomes (Table 4). None of the genotypes suggested the presence of polyspermy. 

### 3.3. Flow Cytometry and DNA Content Analysis

Based on the DNA content analysis, we distinguished two main groups: A small genome size group (SH), with 7.22 ± 0.71 pg DNA content, and a large genome size group (LH), with 11.60 ± 0.84 pg DNA content. The hybrids were divided into five subgroups based on their parents. Estimated genome sizes of the hybrids are shown for the different parent combinations in Table 1. The majority of the tested progenies belonged to the small genome size (SH) group in the case of each parent combination. Although there was no statistically significant difference in mean DNA content among subgroups of the SH group, we identified some outlier individuals with statistically different DNA content, such as the D4AF3A individual (Table 5).

### 3.4. Chromosome Analysis and Ploidy Level in Different Cytometry Class Groups

The chromosome analysis of hybrid individuals confirmed the results of DNA content analysis. Two large groups could be distinguished by their chromosome number: inter-species triploid hybrids (SH) and inter-species pentaploid hybrids (LH). The triploid hybrids were formed by one maternal gamete (2n) of a functional tetraploid female Russian sturgeon and one paternal gamete (1n) from a functional diploid paddlefish. The chromosome numbers of SH varied between 156 ± 4 and 174 ± 10 (Table 5) due to the different numbers of microchromosomes. The pentaploid LH group contained the entire maternal genome (4n = 250 ± 8) and the paternal haploid genome (n = 60). They had 304 ± 6 chromosomes (Table 5). The morphology of microchromosomes was unclear because of their very small size. The paternal haploid cell lines described by Iegorova et al. [18] were also observed in several individuals (PIT ID D52B1B, D4B091, and D4D979) (Table 5).

**Figure 1:**
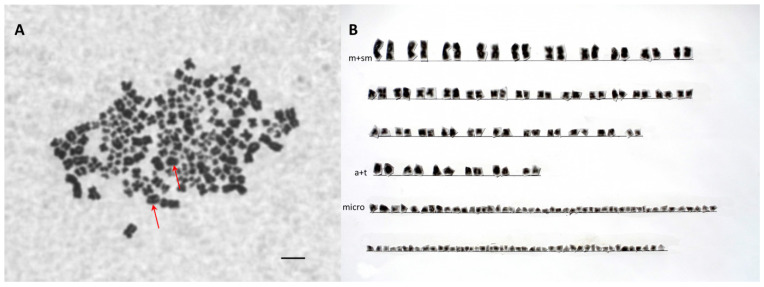
Mitotic metaphase chromosome spread (**A**) from fibroblast cell culture of the pectoral fin of an SH (small genome size hybrids) individual (No. D52B1B). (**B**) Corresponding karyotype of *A. gueldenstaedtii* × *P. spathula* triploid hybrid. Red arrows show the paternal-originated two large acrocentric chromosomes from *P. spathula*. Bar 5 µm.

**Figure 2:**
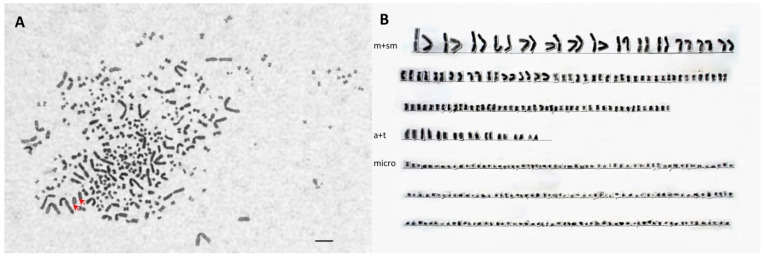
Mitotic metaphase chromosome spread (**A**) from fibroblast cell culture of the pectoral fin of an LH (large genome size hybrids) individual (No. D4D979). (**B**) Corresponding karyotype of *A. gueldenstaedtii.* × *P. spathula.* pentaploid hybrid. Red arrows show the paternal-originated two large acrocentric chromosomes from *P. spathula.* Bar 5 µm.

A detailed karyotype analysis was performed on one randomly chosen SH (PIT ID D52B1B; 3n = 176) and LH individual (PIT ID D4D979; 5n = 310). The karyotype of triploid individuals consisted of 70 meta- and submetacentric, 12 acrocentric, and 94 microchromosomes (Figure 1, Table 6). In the case of pentaploid individuals, the analysis showed the occurrence of 118 metacentric and submetacentric, 18 acrocentric, and 174 microchromosomes (Figure 2, Table 6). In both cases, two large acrocentric chromosomes (Acipenseriformes marker chromosomes) were found (Figure 1 and Figure 2). The reference data of karyotypes found in Table 6 regarding the number of middle-size acrocentric chromosomes were incomplete because references [3,8] did not classify them separately. 

### 3.5. Morphometry

The most pronounced difference between American paddlefish and Russian sturgeon occurred in morphometric characters related to the rostrum length (rc, R, rr, C), which were higher in American paddlefish (Table 3). Russian sturgeons had longer upper caudal lobe (Cfa) and pectoral fin (IP). The rostrum length related characters of hybrids differed from the parent species’, and further differences occurred between the LH and SH groups. The upper caudal lobe (Cfa) and pectoral fin (IP) lengths showed similar patterns, but in the latter character, the LHs differed just marginally from the Russian sturgeons. The size and number of scutes were higher in Russian sturgeon, lower in the SH group, and intermediate in the LH group. The number of lateral scutes was typically higher in hybrids. 

**Figure 3:**
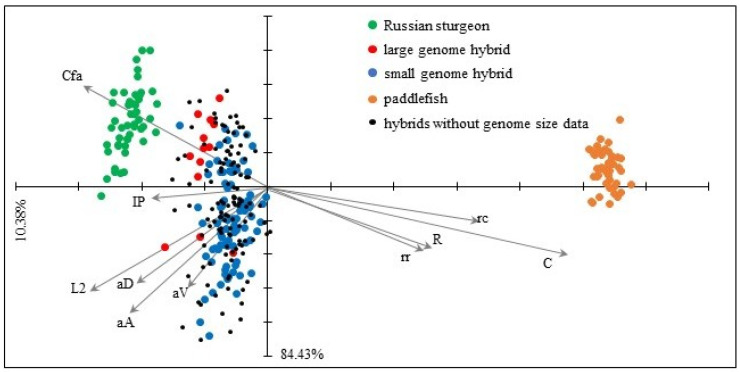
Principal component analysis (PCA) plot for morphometric characters. The percentage of total variance associated with each component is provided. Abbreviations: Cfa: length of the upper caudal lobe, IP: length of pectoral fin, L2: standard length, aD: predorsal distance, aA: preanal distance, aV: preventral distance, rr: snout length, R: preorbital length, C: head length, rc: snout tip–barbel base distance.

**Figure 4:**
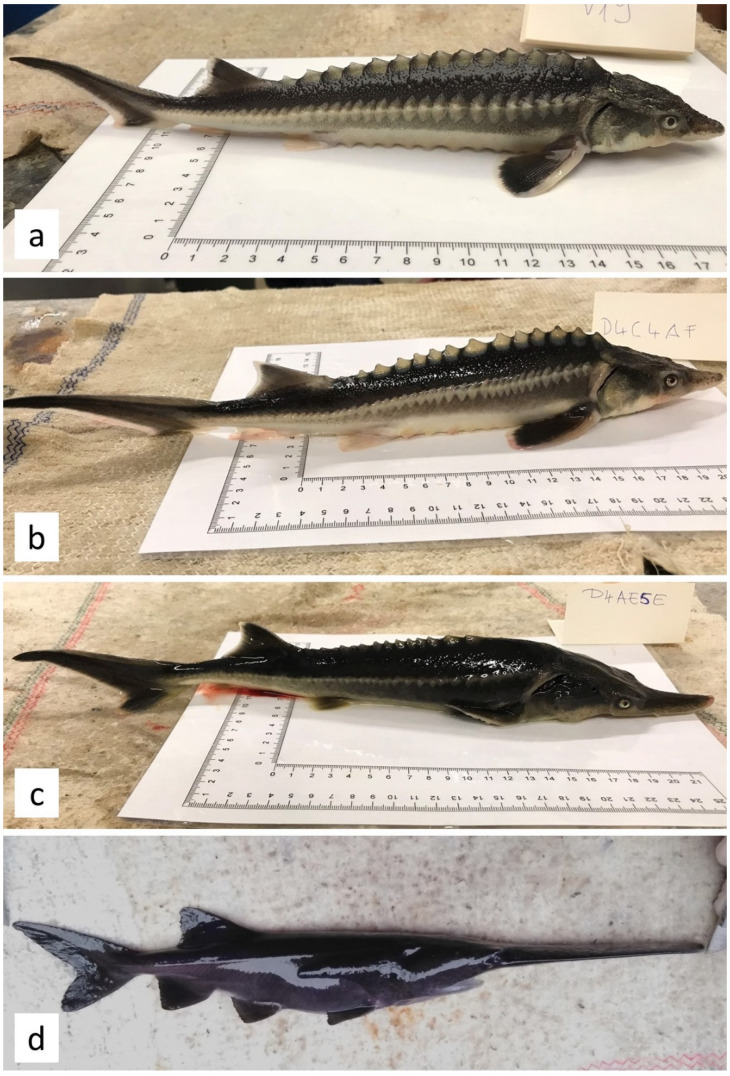
(**a**) Yearlings of *A. gueldenstaedtii* and (**b**) their hybrids: (**c**) typical LH hybrid, (**d**) typical SH hybrid of *P. spathula*.

The first principal component accounted for the vast majority of the total variance (84.43%); the subsequent component explained much less (10.38%; Figure 3). The ordination showed pronounced differentiation between the American paddlefish and Russian sturgeon. The hybrids were closer to the Russian sturgeon. The PCA plot supported the result of the univariate test and highlighted the importance of rostrum length-related characters (rr, R, rc, C) and upper caudal lobe (Cfa) and pectoral fin (IP) length in the separation of hybrids and parent species (Figure 3 and Figure 4). Although we could not divide the hybrids into distinct subgroups, the LH individuals were projected closer to the Russian sturgeon.

## 4. Discussion 

Former experiences on the hybridization of acipenserids and paddlefishes [14,38] support the assumed inability of the hybridization of taxa with such a high phylogenetic distance separating them. Still, the cross of female Russian sturgeon and male American paddlefish, as a negative control for gynogenesis, resulted in viable hybrids. The fertilization, hatching, and survival rates of these hybrids were close to that of the pure maternal species. The progenies exhibited two ploidy levels (triploid and pentaploid) due to spontaneous polyploidization, relating to external morphological and meristic characteristics. 

The reproductive success of these two distant species could be the combined consequence of the ancient genome duplication (180 Mya, that occurred in the common ancestor of the two families) and the slow evolution rate of acipenserids. The theory of slow evolution was reinforced by the “small” morphological differences of the living sturgeon species, the smaller substitution rates of mitochondrial or nuclear genes, and the slower protein evolution, compared to other fish species. This theory has been recently confirmed by a detailed evaluation of the sterlet genome [39]. Since the common sturgeon genome duplication, the sterlet genome has not formed back to a diploid state, and structural and functional elements have been found on the tetraploid level in unexpectedly high degree [39]. These phenomena could lead to a higher similarity, compatibility, and flexibility among the sturgeon genomes and allow the hybridization between Russian sturgeon and American paddlefish despite the large geographical, physiological, and morphological distances.

The intermediate appearance is common in interspecific hybrids [40,41]. A further increase of morphological variability occurs in polyploid hybrids, primarily due to the difference in gene dosage from parent species [42]. Hybrids showed the widest variability in characters, which differed the most parental species, i.e., rostrum-length-related characters and length of the upper caudal lobe. The LH individuals were more similar to the maternal species, presumably due to the larger gene dosage from Russian sturgeon overwhelming the paddlefish-like traits [43]. The SH hybrids showed a more diverse appearance since the ration of maternal and paternal chromosome sets is more balanced than LH hybrids [43]. 

Similar to the interspecific teleost hybrids, where a substantial difference has been observed in size and the number of scales between hybrids and parental species [40,43], the number of scutes differed substantially in hybrids in conjunction with ploidy level. The presence or absence of the scutes, as well as their modified forms in the hybrids, offer the opportunity to study the role of the fibroblast growth factor receptor gene (fgfr) as it influences the number and size of scales in the mirror, nude, and scaled forms of common carp and zebrafish [44]. Sub-functionalization of paralogues of this gene may have occurred following genome duplication during the evolutionary process [45].

The characteristics of karyotypes and the results of microsatellite analysis confirmed that the progenies were interspecies hybrids. As with Váradi et al. [46], chromosome analysis revealed the occurrence of two ploidy levels in hybrids: triploid hybrids (SH, AP) and pentaploid hybrids (LH, AAP). Slight variation in chromosome numbers was also observed within the groups. On the one hand, inconsistency in the published chromosome numbers within a pure-bred species could be the consequence of difficulties in the determination of microchromosome numbers [37]. On the other hand, microchromosome loss also occurs in interspecies hybrids of species characterized by different ploidy levels [47]. We observed haploid paternal cell lineages in some individuals in both groups, suggesting polyspermy [18]. In this case, a supernumerary spermatozoon began to develop as a single cell line, and triploid/pentaploid and haploid cell lines were present in the genome of individuals, causing mosaicism in the hybrids.

Investigations on the functional scale of the ploidy level [20] assume that the interspecific differences in ploidy levels (e.g., diploid American paddlefish and tetraploid Russian sturgeon) originated from whole-genome duplication events during the evolution of Acipenseriformes [8]. Furthermore, intraspecific polyploidization is also relatively frequent among the acipenseriform fishes. It has been detected in white sturgeon (*Acipenser transmontanus*) [48], Siberian sturgeon (*Acipenser baerii*) [20,49], kaluga (*Huso dauricus*) [14], sterlet [50], and Sakhalin sturgeon (*Acipenser mikadoi*) [14]. The spontaneous intraspecific polyploidization can result in variance in genome size. Havelka et al. [20] described the spontaneous duplication of the maternal chromosome set in Siberian sturgeon of different ploidy caused by either premeiotic endomitosis or the retention of the second polar body. Many other studies [51,52,53] have stated the retention that the second polar body can lead to polyploidization. The different ratios of triploid/pentaploid individuals among the parent combinations in our study also supported this hypothesis. 

Microsatellites from both species and hybrids were amplified successfully. All the used microsatellite markers were suitable for distinguishing the two species and for verification of the hybridization. Previously, Zou et al. [14] used the Psp-28, Psp-29, Psp-32 markers on Polyodontidae and Acipenseridae fishes to verify the success of gynogenesis. They also found different allele ranges between the paddlefish and Amur sturgeon. However, the allele sizes could not be compared directly because different techniques were used for allele sizing (polyacrylamide gels and silver staining). All three Psp loci were disomic in that experiment, while the Psp-29 appeared to be tetrasomic in our experiment as well as in the description of Heist et al. [24]. The analyses proved the presence of both the Russian sturgeon and American paddlefish genomes in all examined hybrid individuals, discarding the possibility of spontaneous gynogenesis, which was observed in Siberian sturgeons [20,49] and sterlet [54]. The two tetrasomic markers enabled us to follow the segregation of the tetraploid Russian sturgeon genome and to analyze ploidy levels. These markers showed the presence of only one American paddlefish and two Russian sturgeon alleles in the SH genomes, while in the LH genomes, more alleles were found. This suggested the presence of spontaneous duplication of the maternal genomes in the individuals with high chromosome numbers (250 ± 8–304 ± 4) and/or nucleus size. 

The polyploid status of the species within the Acipenserdae family, as well as their similar karyotype and genome, contribute to easy interspecific and intergeneric hybridization [3]. Based on our findings, successful interspecific hybridization is also supposed in other species combinations in the order Acipenseriformes. Consequently, American paddlefish may hybridize with other sturgeons independent of ploidy level.

The aquaculture potential of this hybrid is a subject for debate. Sturgeon hybrids are commonly used in aquaculture to exploit the advantages of heterosis [55]. It is estimated that hybrids account for approximately 35 and 20% percent of global sturgeon meat and caviar production, respectively [56]. If the planktivorous feeding habit of parent species of paddlefish is inherited to a certain extent, the new hybrid can play an important role in adapting pond aquaculture to the challenges of climate change. Non-fed species have a lower carbon footprint than fed ones, and the co-culture of a filter feeder with relatively high market value as a supplementary species would strengthen pond aquaculture both from an ecological and environmental perspective [57].

## Figures and Tables

**Table 1:** Parents and their progenies with expected and unexpected ploidy groups. Abbreviations: SH: small genome size, LH: large genome size.

Codes of Parents		Progeny with Expected Ploidy				Progeny with Spontaneous Alloploidy			
Female	Male	n	Range (pg)	Mean (pg)	Ploidy	n	Range (pg)	Mean (pg)	Ploidy
6F15	2E75	11	6.96–8.1	7.55 ± 0.39	3n (SH)	1	10.7	-	5n (LH)
1748.	2E75	9	7.19–8.42	7.59 ± 0.33	3n (SH)	3	11.78–12.71	12.09 ± 0.44	5n (LH)
Ag03	596B	22	4.39–7.79	6.56 ± 0.69	3n (SH)	1	10.36	-	5n (LH)
Ag03	551F	24	6.53–8.84	7.62 ± 0.55	3n (SH)	1	13.17	-	5n (LH)
Ag03	6517	17	5.93–8.16	7.05 ± 0.51	3n (SH)	8	10.87–12.87	11.48 ± 0.68	5n (LH)

**Table 2:** Sequence and annealing temperature of microsatellite primers.

Locus	Inheritance		Primer Sequences (5′-3′)	Annealing Temperature (°C)	Repeat Motif
	*P. spathula*	*A. gueldenstaedtii*			
Psp-28	Disomic	Tetrasomic	F: Tail-CAAAGGCATCCCCTACCAC	56	GA
			R: GCTGGACAAAAAGTATGGAGTGC		
Psp-29	Tetrasomic	Disomic	F: Tail-GGGGTCTAATAAAATCCACCGTTC	56	GCAC
			R: TTGCCTTGTGCTCTGTGTTCC		
Psp-32	Disomic	Tetrasomic	F: Tail-AATGACTCAGTTGTGTGCTGC	60	GT
			R: AAGTGTAGGGGAATCTCACCAG		
Spl-101	Disomic	Tetrasomic	F: Tail-CCCTCCACTGGAAATTTGAC	52	TCTA
			R: GCAATCAACAAG GTCTCTTTCA		
Tail (17bp)	-	-	ATTACCGCGGCTGCTGG	-	-

**Table 3:** Mean morphometric (mm) and meristic characteristics among Russian sturgeon and American paddlefish and their hybrids. The numbers in parentheses are the coefficient of variation percentage values. The hybrids were grouped by genome size into small genome hybrids, large genome hybrids, and hybrids without genome size data. Different bold letters (a, b, c, d) indicate the significant differences (*p* > 0.05) based on Kruskal–Wallis tests and Mann–Whitney U pairwise comparisons on standardized data. The data were standardized on the total length; hence, this character was not available for pairwise comparison.

		Russian Sturgeon			Hybrids									American Paddlefish		
					Large Genome			Small Genome			Without Genome Data					
**Morphometric Characters**																
TL	total length	328.10	(11.63)	-	350.27	(31.54)	-	308.52	(24.77)	-	323.79	(25.31)	-	239.45	(10.44)	-
L2	standard length	242.48	(11.46)	**a**	257.93	(30.84)	**b**	235.34	(23.98)	**a**	245.02	(24.19)	**ab**	196.94	(10.82)	**c**
aA	preanal distance	201.78	(11.97)	**a**	221.80	(31.08)	**a**	200.96	(24.31)	**b**	208.93	(24.01)	**b**	169.04	(10.75)	**c**
aD	predorsal distance	191.62	(11.43)	**ab**	204.93	(30.73)	**b**	187.47	(23.37)	**a**	194.07	(23.84)	**ab**	160.18	(10.54)	**c**
aV	preventral distance	161.32	(11.82)	**ab**	173.13	(31.08)	**b**	158.17	(23.72)	**a**	164.31	(23.21)	**a**	145.78	(10.14)	**c**
Cfa	length of upper caudal lobe	92.50	(13.33)	**a**	94.15	(33.65)	**b**	76.81	(28.11)	**b**	83.61	(30.08)	**b**	43.43	(9.58)	**c**
Cfb	length of lower caudal lobe	34.12	(13.85)	**a**	43.48	(36.16)	**b**	37.46	(27.31)	**b**	39.96	(27.15)	**b**	36.31	(10.56)	**c**
C	head length	61.26	(8.81)	**a**	81.97	(27.5)	**b**	84.93	(21.28)	**c**	86.48	(18.79)	**c**	147.43	(9.37)	**d**
R	preorbital length	26.56	(10.29)	**a**	38.30	(26.6)	**b**	44.62	(19.51)	**c**	44.41	(18.59)	**c**	78.99	(9.76)	**d**
IP	length of pectoral fin	41.85	(10.95)	**a**	44.01	(26.08)	**ab**	37.90	(20.29)	**b**	40.46	(21.19)	**b**	18.73	(12.37)	**d**
IV	length of ventral fin	22.60	(13.81)	**a**	28.01	(33.02)	**b**	23.78	(25.21)	**b**	25.51	(25.57)	**b**	14.47	(12.56)	**c**
IA	length of anal fin	26.81	(15.39)	**a**	30.56	(34.13)	**ab**	27.43	(25.46)	**b**	29.60	(25.02)	**b**	22.92	(13.64)	**c**
ID	length of dorsal fin	25.27	(15.31)	**a**	28.53	(33.62)	**ab**	25.91	(24.09)	**b**	27.06	(24.07)	**b**	23.53	(10.76)	**c**
HC	head depth	28.66	(11.64)	**a**	28.46	(28.9)	**b**	26.70	(19.94)	**b**	27.74	(22.64)	**b**	17.43	(10.82)	**c**
h	caudal peduncle depth	11.03	(13.96)	**a**	10.70	(33.36)	**b**	9.87	(27.18)	**b**	10.53	(28.77)	**b**	6.56	(12.88)	**c**
iO	interorbital distance	21.54	(9.77)	**a**	23.58	(24.7)	**a**	24.15	(18.07)	**b**	24.70	(18.57)	**b**	18.76	(8.25)	**c**
rr	snout length	28.46	(9.5)	**a**	40.80	(25.95)	**b**	46.21	(21.66)	**c**	46.04	(19.29)	**c**	79.02	(9.63)	**d**
rc	snout tip–barbel base distance	12.26	(11.71)	**a**	19.52	(27.38)	**b**	26.25	(20.22)	**c**	25.91	(20.37)	**c**	65.84	(9.44)	**d**
SRr	head width on mouth	27.70	(10.47)	**a**	27.63	(23.9)	**b**	27.52	(19.04)	**ab**	27.77	(19.85)	**ab**	20.86	(10.59)	**c**
SRC	head width on barbel base	18.12	(11.82)	**a**	17.90	(21.81)	**a**	19.01	(19.42)	**b**	19.30	(21.61)	**b**	17.10	(8.5)	**c**
SO	mouth width	13.66	(12.58)	**a**	16.32	(28.87)	**b**	17.31	(22.31)	**c**	17.59	(21.09)	**c**	16.64	(11.3)	**d**
O	eye diameter	6.20	(8.93)	**a**	6.79	(13.48)	**a**	6.29	(14.14)	**a**	6.38	(14.85)	**a**	3.80	(11.07)	**b**
sL	scute length	10.76	(11.04)	**a**	9.81	(11.99)	**a**	3.71	(103.77)	**b**	4.77	(62.9)	**b**	-	-	-
Meristic parameters																
sDL	number of dorsal scutes	13.06	(12.71)	**a**	11.8	(40.79)	**a**	3.9	(125.53)	**b**	5.37	(98.16)	**b**	-	-	-
sLL	number of lateral scutes	37.72	(11.56)	**a**	42.6	(10.38)	**b**	45.01	(20.83)	**bc**	47.18	(15.87)	**c**	-	-	-
sVL	number of ventral scutes	10.14	(13.22)	**a**	7.93	(34.18)	**b**	5.01	(45.21)	**c**	5.55	(39.21)	**c**	-	-	-
nB	number of barbel	4	(0)	-	3.47	(28.57)	-	2.76	(40.26)	-	2.87	(36.24)	-	2	(0)	-

**Table 4:** Examples of microsatellite genotypes of SH and LH individuals. (RS: Russian sturgeon; P: paddlefish; SH: hybrid with small genome; LH: hybrid with large genome). Psp-28 was disomic, and Psp-29 was tetrasomic on the paddlefish; Psp-28, Psp-32, and Spl-101 were tetrasomic on Russian sturgeon (paternal alleles are in italics). PIT IDs are the individual numbers of internal tags for individual identification.

PIT ID	Genome Type	Psp-28				Psp-29				Psp-32					Spl_101					
6F15	RS1	220	222			201				134	162	164	166		294		322	330	334	
174B	RS2	220				201					162	164	166		294	304		330		
Ag04	RS3	220				201					162	164	166		294	304		330		
2E7B	P1			*260*			*211*		*225*					*196*						*274*
596B	P2			*260*	*267*		*211*		*225*					*196*						*274*
6517	P3			*260*	*267*		*211*	*218*	*225*					*196*						*274*
D50F80	SH	220		*260*		201	*211*		*225*	134		164		*196*				330	334	*274*
D4BE1D	SH	220		*260*		201	*211*		*225*	134		164		*196*			322	330		*274*
D4B65A	SH	220		*260*		201	*211*		*225*	134		164		*196*			322	330		*274*
D509E7	SH	220		*260*		201	*211*		*225*	134		164		*196*			322	330		*274*
D4B071	SH	220		*260*		201	*211*		*225*	134	162			*196*	294		322			*274*
D4AE5E	SH	220		*260*		201	*211*		*225*		162		166	*196*	294			330		*274*
D49E04	SH	220	222	*260*		201	*211*		*225*		162		166	*196*	294		322			*274*
D4BA3F	SH	220	222	*260*		201	*211*		*225*		162		166	*196*			322	330		*274*
D4A57A	SH	220	222	*260*		201	*211*		*225*		162		166	*196*			322		334	*274*
D4C792	SH	220		*260*		201	*211*		*225*			164	166	*196*	294	304				*274*
D49F88	SH	220		*260*		201	*211*		*225*			164	166	*196*		304				*274*
D4C16A	SH	220			*267*	201		*218*	*225*			164	166	*196*	294	304				*274*
D4C4AF	LH	220	222	*260*		201	*211*		*225*	134	162	164	166	*196*	294		322			*274*
D4D979	LH	220		*260*		201	*211*		*225*	134		164		*196*	294	304		330		*274*
D4C8FF	LH	220		*260*		201	*211*		*225*		162	164	166	*196*	294	304		330		*274*
D4B8EB	LH	220		*260*		201	*211*		*225*		162	164	166	*196*		304		330		*274*
D4B4DF	LH	220		*260*		201	*211*		*225*		162	164	166	*196*	294	304		330		*274*
D50340	LH	220		*260*		201	*211*		*225*		162		166	*196*	294	304		330		*274*
D522C1	LH	220			*267*	201	*211*		*225*		162	164	166	*196*	294	304		330		*274*

**Table 5:** DNA content and chromosome number of the different hybrid types (SH/AP and LH/AAP). AP was an interspecies triploid hybrid with two maternal (4n) genomes and one paternal (2n) genome characterized by small genome size (SH). AAP was an interspecies pentaploid hybrid with four maternal genomes and one paternal genome characterized by large genome size (LH). PIT IDs are the individual numbers of internal tags for individual identification.

PIT ID of Individuals	DNA Content (pg)	Chromosome Number	Remarks
D4AF3A	4.39 ± 0.47	156 ± 4 (3n)	interspecies triploid hybrid (SH/AP)
D4C16A	7.49 ± 0.35	170 ± 2 (3n)	interspecies triploid hybrid (SH/AP)
D4B091	7.57 ± 0.39	170 ± 6 (3n)	interspecies triploid hybrid (SH/AP), paternal haploid cell lineage (1n = 66)
D4AE5E	8.42 ± 0.71	160 ± 4 (3n)	interspecies triploid hybrid (SH/AP)
D52B1B	6.85 ± 0.78	176 ± 8 (3n)	interspecies triploid hybrid (SH/AP), paternal haploid cell lineage (1n = 66–68)
D4D979	10.87 ± 0.65	308 ± 4 (5n)	pentaploid hybrid (LH/AAP), dicentric chromosome was found, paternal haploid cell lineage (1n = 68)
D4C9E4	12.36 ± 0.84	302 ± 4 (5n)	pentaploid hybrid (LH/AAP)
D522C1	12.87 ± 1.58	306 ± 4 (5n)	pentaploid hybrid (LH/AAP)
*Polyodon spathula*	3.90	120 (2n)	Symonová et al. [8]
*Acipenser gueldenstaedtii*	7.86–7.88	258 ± 4 (4n)	Fontana et al. [37]

**Table 6:** Characteristics of karyotypes of triploid and pentaploid hybrids compared with parental species (based on Birnstein et al.) [3].

Species/PIT ID	Number of Metacentric Plus Submetacentric Chromosomes	Number of Middle-Sized Acrocentric Chromosomes	No. of Large Acrocentric Chromosomes	Number of Microchromosomes	Total Chromosome Number
*Polyodon spatula*	44		4	72	120 (PP)
*Acipenser gueldenstaedtii*	92		-	155	247 (AA)
D52B1B	70	10	2	94	176 (SH-AP)
D4D979	118	16	2	174	310 (LH-AAP)
